# Algorithm for an automatic treatment planning system using a single‐arc VMAT for prostate cancer

**DOI:** 10.1002/acm2.13442

**Published:** 2021-10-08

**Authors:** Takumi Kodama, Shigehiro Kudo, Shogo Hatanaka, Masatsugu Hariu, Munefumi Shimbo, Takeo Takahashi

**Affiliations:** ^1^ Department of Radiation Oncology Ina Saitama Prefectural Hospital Organization Saitama Cancer Center Saitama Japan; ^2^ Department of Radiation Oncology Saitama Medical Center Saitama Medical University Kawagoe Saitama Japan

**Keywords:** automated treatment planning, Monaco, optimization, prostate, VMAT

## Abstract

Optimization process in treatment planning for intensity‐modulated radiation therapy varies with the treatment planner. Therefore, a large variation in the quality of dose distribution is usually observed. To reduce variation, an automatic optimizing toolkit was developed for the Monaco treatment planning system (Elekta AB, Stockholm, Sweden) for prostate cancer using volumetric‐modulated arc therapy (VMAT). This toolkit was able to create plans automatically. However, most plans needed two arcs per treatment to ensure the dose coverage for targets. For prostate cancer, providing a plan with a single arc was advisable in clinical practice because intrafraction motion management must be considered to irradiate accurately. The purpose of this work was to develop an automatic treatment planning system with a single arc per treatment for prostate cancer using VMAT. We designed the new algorithm for the automatic treatment planning system to use one arc per treatment for prostate cancer in Monaco. We constructed the system in two main steps: (1) Determine suitable cost function parameters for each case before optimization, and (2) repeat the calculation and optimization until the conditions for dose indices are fulfilled. To evaluate clinical suitability, the plan quality between manual planning and the automatic planning system was compared. Our system created the plans automatically in all patients within a few iterations. Statistical differences between the plans were not observed for the target and organ at risk. It created the plans with no human input other than the initial template setting and system initiation. This system offers improved efficiency in running the treatment planning system and human resources while ensuring high‐quality outputs.

## 1 INTRODUCTION

Intensity‐modulated radiation therapy (IMRT) and volumetric‐modulated arc therapy (VMAT) are now standard techniques for radiation therapy in clinical practice. Inverse treatment planning for these techniques involves the optimization of an intensity map and leaf sequences. The treatment planner needs to set parameters and optimize several times until clinically acceptable plans are obtained. The parameter settings and the number of iterations vary with the treatment planner. Therefore, a large variety of dose distributions exist despite these doses staying within the goal.^1^, ^2^, ^3^, ^4^ Moreover, treatment planning is labor‐intensive work. The optimization process undergoes trial and error because the relationship between most of the parameters and the dose distribution is unclear.

In recent years, an automatic mode in the treatment planning system (TPS) has been implemented. This automation reduces interplanner variation and TPS operating time.^5^, ^6^, ^7^ The Monaco TPS version 5.1.0.4 (Elekta AB, Stockholm, Sweden) has not been implemented as an automatic planning system. Therefore, Ayala et al.^8^ developed an automatic optimizing toolkit named Pymonaco. Pymonaco was written in the Python language and open‐accessible to the public. This toolkit can automatically adjust parameters such as the degree of fluence smoothing or the maximum number of arcs per treatment based on the information in a text file with the extension “.hyp.” This file contains optimization settings and results, such as cost function, “shrink margin,” weight, and “isoconstraint” for each cost function, and “isoeffect.” A “shrink margin” creates a contour smaller than the original volume by a specified distance in order to deal with abutting structures or structures, which are close to each other. An “isoconstraint” is the objective value for each cost function. An “isoeffect” is the value corresponding to the isoconstraint in the current dose distribution. Ayala et al.^8^ implemented their auto flowchart for prostate cancer using VMAT. They started the calculation for dose distribution with an initial template using a single arc per treatment. If the dose coverage for the planning target volume (PTV) is not satisfied by the threshold value, Pymonaco recalculated using two arcs and set the fluence smoothing level to be relaxed.

Ayala et al.^8^ chose the maximum number of arcs first to improve dose distribution and reported that most treatment plans were created with two arcs per treatment. Two arcs can certainly generate a more complex dose distribution than a single arc can.^9^ On the other hand, the displacement of the prostate increased with elapsed time after image‐guided positioning, and motion during treatment produced several patterns from individual patients or daily treatment.^10^, ^11^, ^12^ Therefore, short radiation delivery time and the initiation of treatment delivery as soon as possible following image‐guided positioning are important.

Thus, the ability of the planner to provide a satisfactory plan in clinical practice with a single arc per treatment for prostate cancer is important. However, no auto flowchart using a single arc for prostate cancer using VMAT for Monaco exists. The present work attempted to address this gap with the development of an automatic TPS with a single arc per treatment using VMAT for prostate cancer and evaluate the clinical suitability of this system for treatment planning.

## 2 MATERIALS AND METHODS

### 2.1 Patient selection

Twenty prostate patients treated in our institution were selected randomly for the retrospective study with approval from the institutional review board.

### 2.2 Treatment planning system

We calculated all plans using Monaco version 5.1.0.4 based on our clinical dose constraint. The dose plans were calculated using the X‐ray Voxelized Monte Carlo algorithm with a dose grid resolution of 2 mm. The plans were made using a model for a Varian Novalis‐Tx linear accelerator (Varian Medical Systems, Inc., Palo Alto, CA, USA) for 6 MV photons. Within Monaco, the user must select the planning template for creating new plans. The template contains the information required in the planning process as follows: beam setup and geometry (beam model, energy, gantry angle, collimator angle, number of beams, arc increment, etc.), prescription parameters (prescription dose, number of fractions, etc.), optimization parameters (cost function settings, beamlet width, maximum number of control points per arc, minimum segment width, fluence smoothing, etc.), and dose calculation settings (grid size, dose deposition media, statistical uncertainty, etc.). The main stable parameters and settings are shown in Table 1. For inverse planning, Monaco has two optimization modes: constrained and pareto.^13^ In this study, we selected the constrained mode, which prioritizes constraints on organ at risk (OAR) than target coverage. All plans were created using the same initial template to start optimization with a single‐arc VMAT technique and adjusted only the cost function settings indicated in Table 2 in manual planning and the automatic planning system.

**TABLE 1:** List of the main stable parameters and settings

Beam setup and geometry	
Energy	6 MV
Collimator angle	10°
Arc increment	35°
Optimization parameters	
Max number of arcs	1
Max number of control points per arc	160
Minimum segment width	0.80 cm
Fluence smoothing	High
Beamlet width	0.20 cm
Target margin	Narrow (3–4 mm)
Dose calculation settings	
Grid size	2 mm
Dose deposition media	Medium
Statistical uncertainty	0.3% (per calculation)

**TABLE 2:** List of cost function settings in the initial template

Structure name	Cost function	Weight adjust	Weight	Threshold	Isoconstraint	Additional settings
Urethra	Target EUD	Manual	15		7840 cGy	Cell sensitivity: 0.90
	Maximum dose	Manual	30		8050 cGy	
CTV	Underdose DVH	Manual	100	7810 cGy	99%	Optimize over all voxels in volume: yes
	Target penalty	Manual	100		7800 cGy	Minimum Volume: 99%
						Optimize over all voxels in volume: yes
PTV1	Underdose DVH	Manual	100	7800 cGy	99%	
	Target penalty	Manual	100		7800 cGy	Minimum volume: 96%
PTV2	Underdose DVH	Manual	100	7200 cGy	95%	
	Target penalty	Manual	100		7200 cGy	Minimum volume: 96%
	Quadratic overdose	Auto		7810 cGy	9 cGy	
	Overdose DVH	Auto		7400 cGy	60%	
zRectum	Serial	Auto			800 cGy	Power law exponent: 20
	Quadratic overdose	Auto		7000 cGy	9 cGy	
	Quadratic overdose	Auto		2340 cGy	20 cGy	
Bladder	Serial	Manual	20		5000 cGy	Power law exponent: 1
						Shrink margin: 1 cm
	Serial	Manual	20		5000 cGy	Power law exponent: 15
						Shrink margin: 2 cm
Femoral heads	Maximum dose	Manual	20		5000 cGy	
Patient	Quadratic overdose	Auto		7800 cGy	2 cGy	Shrink margin: 0.1 cm
	Maximum dose	Manual	4		7980 cGy	Optimize over all voxels in volume: Yes

EUD, equivalent uniform dose; DVH, dose volume histogram; CTV, clinical target volume; PTV1, area of the planned target volume (PTV) minus the rectum; PTV2, area of the overlap of the rectum with the PTV; zRectum, adding 5 mm margins to the rectum in the posterior direction and the subtract volume was created by adding a 3 mm margin to the PTV.

### 2.3 Prescription and structure contouring

The clinical target volume (CTV) was contoured by the radiological oncologist. The PTV was created by adding 8 mm margins to the CTV in all directions, except in the posterior direction, where a 5 mm margin was added to the CTV. PTV1 was the area of the PTV minus the rectum, and PTV2 was the area of overlap of the rectum with the PTV. The prescription protocol was that 95% of the volume was received in 39 treatment fractions as 78 and 72 Gy for PTV1 and PTV2, respectively. All plans were normalized such that 95% of the PTV1 received the prescription dose. In addition to the target, the OAR was delineated as the rectum, bladder, urethra, and femoral heads. To control dose distribution, a dummy volume named zRectum was created by adding 5 mm margins to the rectum in the posterior direction, and the subtract volume was created by adding a 3 mm margin to the PTV1.

### 2.4 Automatic treatment planning system

The automatic TPS was implemented using Python version 3.6.8. Additional Python modules Pywinauto^14^ version 0.6.6 and PyInstaller^15^ version 3.4 were used in our system. Pywinauto is a Python module for automating the Microsoft Windows graphical user interface. It allows the user to send mouse and keyboard actions to Windows dialogs and controls. PyInstaller is a Python module for bundling a Python application and all its dependencies into a single package. The user can run the packaged application without installing a Python interpreter or any modules.

An executable package file of the automatic planning system was created with PyInstaller and installed on the desktop in Monaco. This package file contained all Python applications required to execute the automatic planning system. Moreover, the system enabled the planner or task scheduler to set it to automatically perform at a chosen time on the Windows system.

**FIGURE 1:**
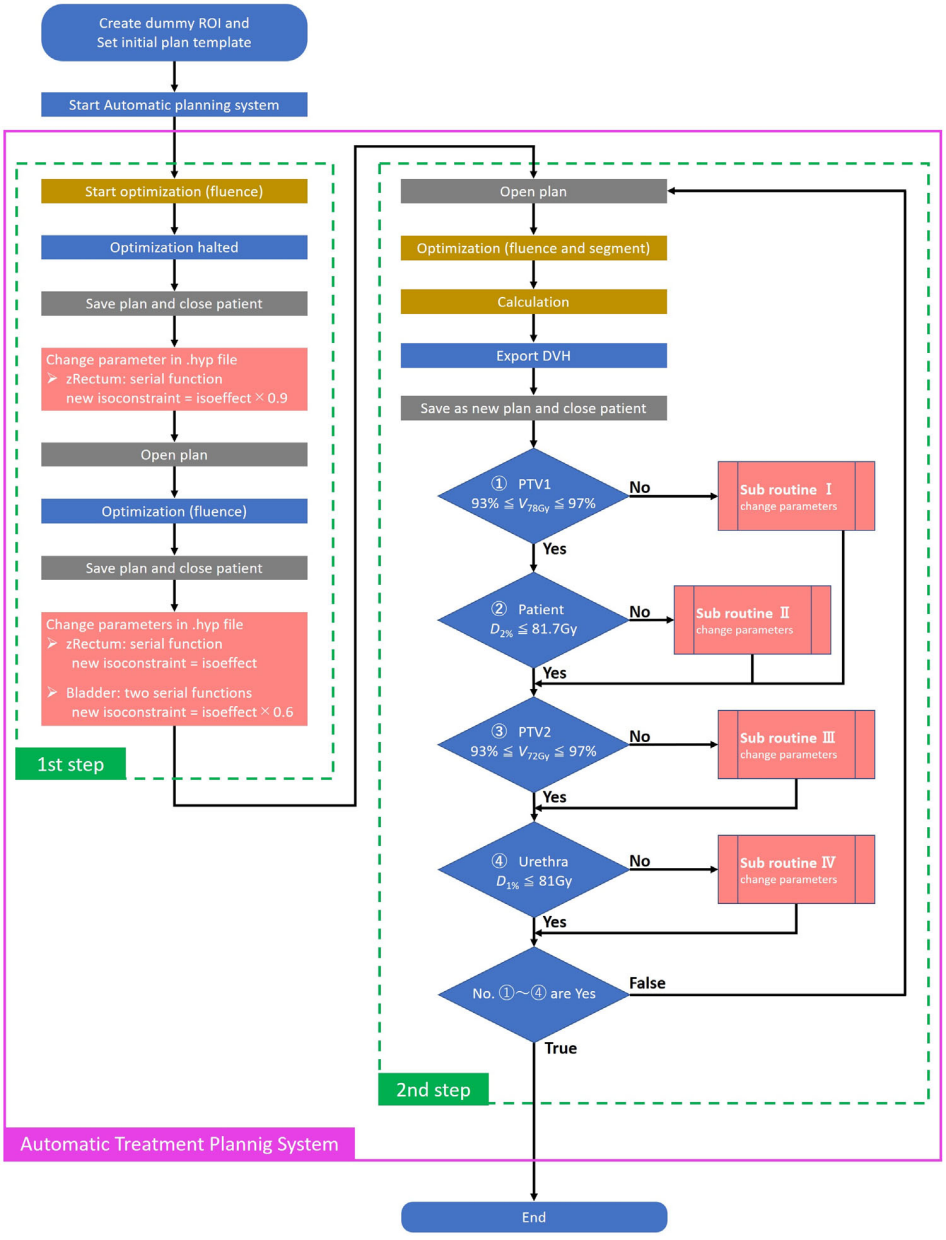
Flowchart of the automatic treatment planning system

We constructed the system in two main steps, as illustrated in the flowchart in Figure 1. The planner started the automatic treatment planning system after the “preparation” process (see the next section). The first step is performed to determine suitable isoconstraint values for zRectum and bladder. The second step iterated parameter adjustments to fulfill all conditions. The preparation and algorithms of these steps are described in detail in the sections that follow.

#### 2.4.1 Preparation

Before starting the automatic planning system, the planner needs to create the dummy volume and set the plan template. In our institution, treatment plans were created by the medical physicist, after which the radiological oncologist contoured the target and OAR. For safety, this automatic planning system cannot be used without validating the contours before planning.

#### 2.4.2 First step

Before iterating optimization and adjusting parameters, the first step is to determine the suitable cost function parameters for zRectum and the bladder for each patient. Fluence optimization was halted deliberately by setting too low the isoconstraint serial function values for the zRectum structure in the plan template. Monaco stops the optimization immediately and then detects the unattainable goal. This isoconstraint value was updated to the value of the isoeffect decreased by 10%. Fluence optimization was started again. When fluence optimization had finished, the isoconstraint value was updated again to the isoeffect value. At the same time, the isoconstraints of two serial functions for the bladder were updated to the isoeffect value decreased by 40%.

#### 2.4.3 Second step

Dose distribution was calculated using the parameters set in the first step. The system iterated the optimization and adjusted the parameters to derive an acceptable plan that generated dose indices that fulfilled the following four conditions:

**Table:** 

1. PTV1	: 93% ≤ *V*_78Gy_ ≤ 97%
2. Patient (body)	: *D*_2%_ ≤ 81.7 Gy
3. PTV2	: 93% ≤ *V*_72Gy_ ≤ 97%
4. Urethra	: *D*_1%_ ≤ 81 Gy

where *V_x_*_Gy_ indicates the percentage volume of the contour received *x* Gy and *D_y_*_%_ indicates that y% of the volume of the contour received that dose or more. The system exported a dose volume histogram (DVH) from Monaco to evaluate dose indices after the calculation for dose distribution. If the plan did not fulfill each condition, optimization parameters were adjusted in subroutines I, II, III, and IV. Only the condition for the patient (Condition No. 2) was checked after achieving an adequate dose coverage for PTV1 (Condition No. 1). Figure 2 shows the flowchart for subroutines I, II, III, and IV. In subroutines I and II, the weight of the target dose objectives was increased until the upper limit of 500 or 1000 was reached to achieve adequate dose coverage. If dose coverage was not achieved at the upper limit of the weight, optimization parameters such as weight, isoconstraint, and shrink margin for OAR were adjusted to loosen the direction. The shrink margin is a geometric parameter of the cost function for OAR in Monaco. It allows user‐controlled dose gradients without additional structures by specifying shrink distances from targets when OAR overlaps with the target. On the other hand, subroutines II and IV were simple algorithms that increased the weight of the maximum dose for each structure to decrease the hotspot.

**FIGURE 2:**
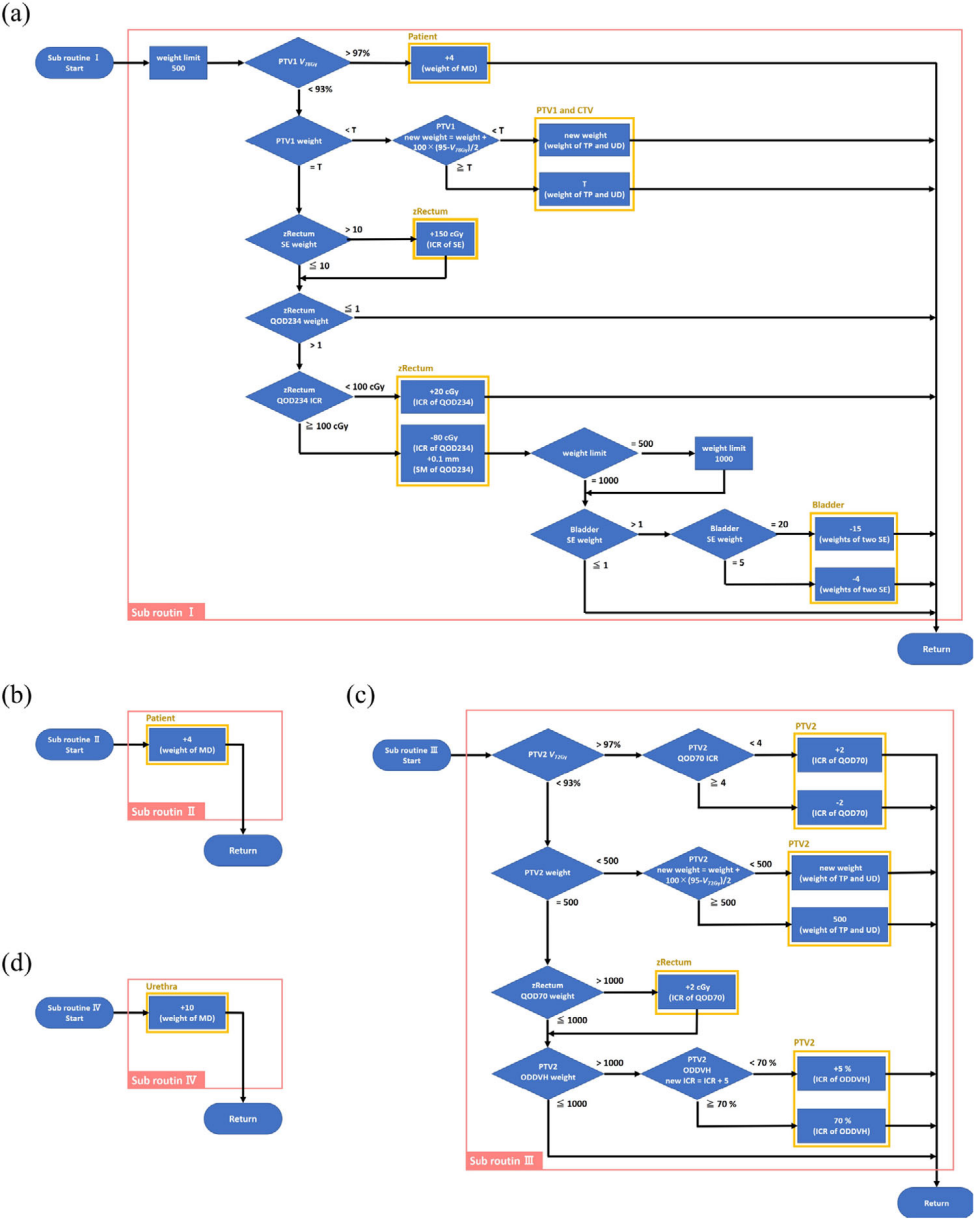
Flowchart of (a) subroutine I, (b) subroutine II, (c) subroutine III, and (d) subroutine IV in the second step of the automatic treatment planning system. MD, maximum dose; DVH, dose volume histogram; UD, underdose DVH; TP, target penalty; SE, serial; QOD234, quadratic overdose set weight on 2340 cGy; QOD70, quadratic overdose set weight on 7000 cGy; ICR, isoconstraint; SM, shrink margin; ODDVH, overdose DVH

### 2.5 Plan comparison and statistical analysis

The plan quality between manual planning and automatic planning was compared using the heterogeneity index (HI)^12^ and conformity index (CI)^16^, ^17^ for PTV1 as follows:

(1) $$HI=D_{1\%}/D_{95\%},CI=T{V_{PD}}^{2}/\left(TV\times V_{PD}\right),$$

where *TV_PD_* is the target volume covered by the prescribed dose, *TV* is the target volume, and *V_PD_* is the volume enclosed by the prescribed isodose surface. Furthermore, we obtained monitor units (MU) and the resulting dose indices for the PTV1 (*D*_2%_ and *D*_98%_), PTV2 (*D*_95%_), rectum (*V*_70Gy_, *V*_65Gy_, and *V*_40Gy_), bladder (*V*_70Gy_, *V*_65Gy_, and *V*_40Gy_), urethra (*D*_2%_), and each femoral head (*D*_1cc_). Further statistical analysis was performed with a paired *t‐*test. The *p* value was calculated with Scipy version 1.2.1,^18^ and graphical representations were performed with Matplotlib version 3.0.3^19^ modules. A *p* value < 0.05 was considered statistically significant.

## 3 RESULTS

**FIGURE 3:**
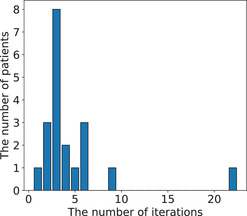
Histogram of the number of iterations until all four conditions are fulfilled by the automatic treatment planning system

**FIGURE 4:**
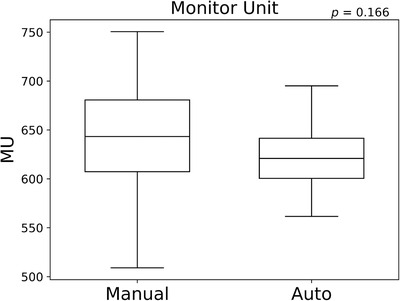
The box and whisker plots of the monitor unit for manual planning and the automatic treatment planning system. The *p* values of the paired *t*‐test between manual planning and the automatic treatment planning system are shown in each figure

The automatic planning system created plans to fulfill all four conditions for all patients. Figure 3 shows a histogram of the number of iterations until all four conditions were fulfilled by the automatic planning system. The median was three iterations, and almost all plans were created within nine iterations, except one. In this case, the system iterated the parameter adjustment many times to fulfill the condition for urethra. Figure 4 shows a boxplot of the MU. The MU were 643.1 ± 64.4 and 623.2 ± 39.4 for manual planning and the automatic planning system, respectively (*p* = 0.166).

### 3.1 Planning target volume

**FIGURE 5:**
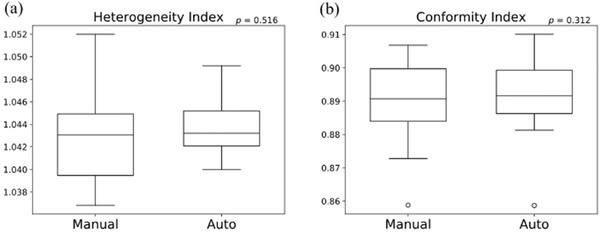
The box and whisker plots of (a) the heterogeneity index and (b) conformity index for manual planning and the automatic treatment planning system. The *p* values of the paired *t*‐test between manual planning and the automatic treatment planning system are shown in each figure

**FIGURE 6:**
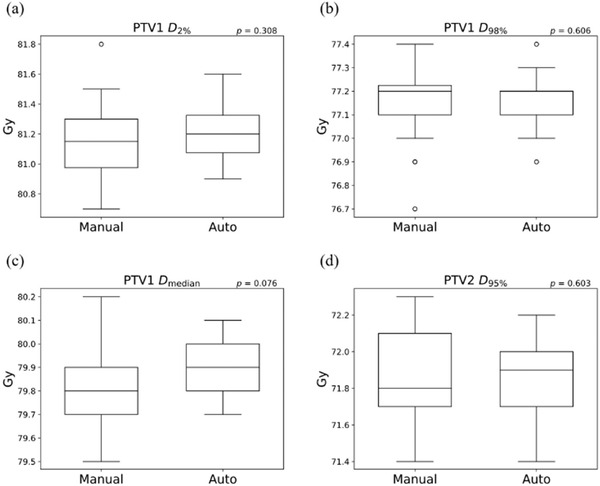
The box and whisker plots of (a) PTV1 *D*_2%_, (b) PTV1 *D*_98%_, (c) PTV1 *D*_median_, and (d) PTV2 *D*_95%_ for manual planning and the automatic treatment planning system. The *p* values of the paired *t*‐test between manual planning and the automatic treatment planning system are shown in each figure

All plans were normalized such that 95% of the PTV1 received the prescription dose of 78 Gy. Figure 5 shows the boxplots of the HI and CI. The HI were 1.043 ± 0.004 and 1.044 ± 0.003 for manual planning and the automatic planning system, respectively (*p* = 0.516). The CI were 0.890 ± 0.013 and 0.892 ± 0.011 for manual planning and the automatic planning system, respectively (*p* = 0.312). Figure 6 shows the boxplot of the DVH indices for PTV. No significant difference was observed between manual planning and the automatic planning system. At PTV1, *D*_median_ was slightly higher using the automatic planning system, but the difference was not statistically significant (*p* = 0.076). The automatic planning system created the prescription dose of 72 Gy for PTV2 with a normalization dose for PTV1.

### 3.2 Organ at risk

**FIGURE 7:**
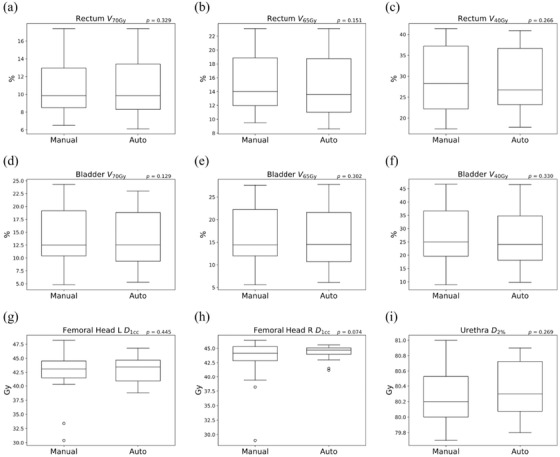
The box and whisker plots of (a) rectum *V*_70Gy_, (b) rectum *V*_65Gy_, (c) rectum *V*_40Gy_, (d) bladder *V*_70Gy_, (e) bladder *V*_65Gy_, (f) bladder *V*_45Gy_, (g) femoral head left *D*_1cc_, (h) femoral head right *D*_1cc_, and (i) urethra *D*_2%_ for manual planning and the automatic treatment planning system. The *p* values of the paired *t*‐test between manual planning and the automatic treatment planning system are shown in each figure

Figure 7 shows the boxplot of the DVH indices for the rectum, bladder, urethra, and femoral heads. Similarly, with PTV, no significant difference was observed between manual planning and the automatic planning system in OAR. For the right femoral head, *D*_1cc_ was slightly higher using the automatic planning system, but the difference was not statistically significant (*p* = 0.074).

## 4 DISCUSSION

A large variation in dose distribution quality has been reported with IMRT.^1^, ^2^, ^3^ This quality depends not on the certification, education demographics, experience, or confidence level of the planners but on their knowledge of adequate techniques.^4^ The automatic planning system has the advantage of creating plans independent of the treatment planner's knowledge of the optimal techniques. Moreover, treatment planning with IMRT has a very large solution space. Therefore, it is labor‐intensive work for the planner. Automating the planning process additionally reduces the amount of time spent on planning.

Our system created the plans automatically for all patients with prostate cancer using a single arc. It indicated that original functions, such as deciding the suitable parameters for each patient in first step and adjusting parameters based on information from DVH were performed well. Constraints for OAR were set strictly to be achieved in the initial template and adjusted to loosen the direction to fulfill the four conditions. Plans were created after a few tries for most of the patients. Therefore, the parameters in the initial template and parameter adjustments by our algorithm were adequate. However, for one patient, the first plan calculated from the initial template fulfilled all four conditions without iterating parameter adjustments. In that case, dose distribution could be possibly improved by implementing additional algorithms, such as adjusting parameters for OAR, to tighten the direction. Additionally to that case, for one patient, the system iterated parameter adjustment many times to fulfill one condition because the parameter was adjusted step by step in our system. To fulfill the conditions in few trials, the system should implement an algorithm that decides the amount of adjustment for the urethra after few trials based on the relation between dose indices and the amount of adjustment.

If our algorithm cannot achieve all four conditions, adjusting the number of arcs is the next option to improve the dose distribution. Because treatment time increases significantly with the number of arcs, it is not a suitable parameter for treatment sites that should be considered in intrafraction motion, such as the prostate. Only three parameters were used in our system, namely, isoconstraint, weight, and shrink margin. Other parameters for improving the dose distribution in a text file with the extension “.hyp” include the number of control points, minimum segment width, beamlet width, and fluence smoothing. To improve the dose distribution, the planner should try adjusting these parameters first. If that does not work, the planner should discuss increasing the number of arcs with the radiological oncologist.

In previous reports, the automatic planning system could further reduce the dose for OAR and/or deliver a homogeneous dose to the target than manual planning could.^5^, ^6^, ^7^, ^8^, ^20^ In the present work, dose distributions created by the automatic TPS had the same quality as manual planning performed by an experienced planner. Because dose distribution has a large variation in quality independent of the planner and the development of treatment planning skills is continually ongoing within any institution, comparing the dose distribution between manual planning and the automatic planning system is inherently difficult, given the possibility that results might vary from one institution to another. Indeed, the automatic planning system has the potential to create better plans. Therefore, the algorithm needs to be improved continuously. Moreover, this algorithm was developed only for prostate. In future investigations, new generalized algorithms, such as that identify whether a cost function belongs to the target or OAR, adjust parameters adequately for the target and OAR, and set conditions for planning a goal, are needed to deal with another treatment sites. Additionally, most important thing is that expert planners are needed to develop these algorithms quickly because the planner should make suitable choices from numerous parameters and adjust that, and understanding the relationship between parameters and dose indices is difficult for beginner treatment planning.

In addition to the plan quality of each patient, the smaller variations were observed in HI and dose indices for PTV in automated plans. As in the previous report,^5^ the automatic planning system might create more consistent plan quality without interplanner variability. Moreover, our system stored all plans until fulfilled conditions, the planner can understand the relationship between each parameter and dose indices. These features have an advantage especially for planners who have never used Monaco. However, the planner can simultaneously create acceptable plans without understanding the treatment planning. Even if the treatment planning system implements an automatic planning system, all planners should continuously develop their treatment planning skills.

Finally, our algorithm created the plans with no human input other than setting the initial template and initiating the automatic planning system. Thus, our algorithm improved the efficiency of using the TPS and human resources while ensuring high‐quality outputs.

## 5 CONCLUSION

We demonstrated a new algorithm that used one arc per treatment for prostate in the Monaco TPS. This algorithm created plans that showed the same quality as that of manual planning within a few iterations of optimization. It reduced variation in the quality of dose distribution and streamlined the treatment planning process while ensuring high‐quality outputs. Future investigations should include a new algorithm that creates better plans in less repeat times for all treatment sites.

## CONFLICT OF INTEREST

The authors declare no conflict of interest.

## AUTHOR CONTRIBUTIONS

Conception and design of study: Takumi Kodama, Shigehiro Kudo, Shogo Hatanaka, Masatsugu Hariu, Munefumi Shimbo, and Takeo Takahashi. Acquisition of data and analysis: Takumi Kodama and Shigehiro Kudo. Interpretation of data: Takumi Kodama, Shigehiro Kudo, Shogo Hatanaka, Masatsugu Hariu, Munefumi Shimbo, and Takeo Takahashi. Drafting the manuscript: Takumi Kodama. Revising the manuscript critically for important intellectual content: Takumi Kodama, Shigehiro Kudo, Shogo Hatanaka, Masatsugu Hariu, Munefumi Shimbo, and Takeo Takahashi. Approval of the version of the manuscript: Takumi Kodama, Shigehiro Kudo, Shogo Hatanaka, Masatsugu Hariu, Munefumi Shimbo, and Takeo Takahashi

## References

[1] Williams MJ , Bailey MJ , Forstner D , Metcalfe PE . Multicenter quality assurance of intensity modulated radiation therapy plans: a precursor to clinical trials. Australas Radiol. 2007; 51(5): 472‐479. 17803801 10.1111/j.1440-1673.2007.01873.x

[2] Chung HT , Lee B , Park E , Lu JJ , Xia P . Can all centers plan intensity‐modulated radiotherapy (IMRT) effectively? An external audit of dosimetric comparisons between three‐dimensional conformal radiotherapy and IMRT for adjuvant chemoradiation for gastric cancer. Int J Radiat Oncol Biol Phys. 2008; 71(4): 1167‐1174. 18234440 10.1016/j.ijrobp.2007.11.040

[3] Batumalai V , Jameson MG , Forstner DF , Vial P , Holloway LC . How important is dosimetrist experience for intensity modulated radiation therapy? A comparative analysis of a head and neck case. Pract Radiat Oncol. 2013; 3(3): e99‐e106. 24674377 10.1016/j.prro.2012.06.009

[4] Nelms BE , Robinson G , Markham J , et al. Variation in external beam treatment plan quality: an inter‐institutional study of planners and planning systems. Pract Radiat Oncol. 2012; 2(4): 296‐305. 24674168 10.1016/j.prro.2011.11.012

[5] Nawa K , Haga A , Nomoto A , et al. Evaluation of a commercial automatic treatment planning system for prostate cancers. Med Dosim. 2017; 42(3): 203‐209. 28549556 10.1016/j.meddos.2017.03.004

[6] Hazell I , Bzdusek K , Kumar P , et al. Automatic planning of head and neck treatment plans. J Appl Clin Med Phys. 2016; 17(1): 272‐282. 26894364 10.1120/jacmp.v17i1.5901 PMC5690191

[7] Chanyavanich V , Das SK , Lee WR , Lo JY . Knowledge‐based IMRT treatment planning for prostate cancer. Med Phys. 2011; 38(5): 2515‐2522. 21776786 10.1118/1.3574874

[8] Ayala R , Ruiz G , Valdivielso T . Automatizing a nonscripting TPS for optimizing clinical workflow and reoptimizing IMRT/VMAT plans. Med Dosim. 2019; 44(4): 409‐414. 30952384 10.1016/j.meddos.2019.02.006

[9] Quan EM , Li X , Li Y , et al. A comprehensive comparison of IMRT and VMAT plan quality for prostate cancer treatment. Int J Radiat Oncol Biol Phys. 2012; 83(4): 1169‐1178. 22704703 10.1016/j.ijrobp.2011.09.015 PMC3805837

[10] Ghilezan MJ , Jaffray DA , Siewerdsen JH , et al. Prostate gland motion assessed with cine‐magnetic resonance imaging (CINE‐MRI). Int J Radiat Oncol Biol Phys. 2005; 62(2): 406‐417. 15890582 10.1016/j.ijrobp.2003.10.017

[11] Kupelian P , Willoughby T , Mahadevan A , et al. Multi‐institutional clinical experience with the calypso system in localization and continuous, real‐time monitoring of the prostate gland during external radiotherapy. Int J Radiat Oncol Biol Phys. 2007; 67(4): 1088‐1098. 17187940 10.1016/j.ijrobp.2006.10.026

[12] Langen KM , Willoughby TR , Meeks SL , et al. Observations on real‐time prostate gland motion using electromagnetic tracking. Int J Radiat Oncol Biol Phys. 2008; 71(4): 1084‐1090. 18280057 10.1016/j.ijrobp.2007.11.054

[13] Clements M , Schupp N , Tattersall M , Brown A , Larson R . Monaco treatment planning system tools and optimization processes. Med Dosim. 2018; 43(2): 106‐117. 29573922 10.1016/j.meddos.2018.02.005

[14] Mahon MM , Herrman M , Intel Corporation, Open Source Community. Pywinauto—Windows GUI automation with Python 2019. https://github.com/pywinauto/pywinauto

[15] Bajo G , PyInstaller Development Team. PyInstaller—freeze (package) python programs into stand‐alone executables 2020. https://github.com/pyinstaller/pyinstaller

[16] van't Riet A , Mak AC , Moerland MA , Elders LH , van der Zee W . A conformation number to quantify the degree of conformality in brachytherapy and external beam irradiation: application to the prostate. Int J Radiat Oncol Biol Phys. 1997; 37(3): 731‐736. 9112473 10.1016/s0360-3016(96)00601-3

[17] Feuvret L , Noël G , Mazeron JJ , Bey P . Conformity index: a review. Int J Radiat Oncol Biol Phys. 2006; 64(2): 333‐342. 16414369 10.1016/j.ijrobp.2005.09.028

[18] Oliphant TE . Python for scientific computing. Comput Sci Eng. 2007; 9(3): 10‐20.

[19] Caswell TA , Droettboom M , Hunter J , et al. Matplotlib/Matplotlib V3.3.1 2020. 10.5281/zenodo.3984190

[20] Creemers IHP , Kusters JMAM , van Kollenburg PGM , Bouwmans LCW , Schinagl DAX , Bussink J . Comparison of dose metrics between automated and manual radiotherapy planning for advanced stage non‐small cell lung cancer with volumetric modulated arc therapy. Phys Imaging Radiat Oncol. 2019; 9: 92‐96. 33458432 10.1016/j.phro.2019.03.003 PMC7807870

